# Case report: Two novel compound heterozygous variant of *SLC12A3* gene in a gitelman syndrome family and literature review

**DOI:** 10.3389/fgene.2024.1391015

**Published:** 2024-07-11

**Authors:** Xiaochen Ji, Nan Zhao, Haixia Liu, Yutong Wu, Lichao Liu

**Affiliations:** ^1^ Department of Internal Medicine, Dalian Medical University, Dalian, China; ^2^ Department of Endocrinology and Metabolism, the Second Affiliated Hospital of Dalian Medical University, Dalian, Liaoning, China

**Keywords:** gitelman syndrome, diabetes mellitus, hypokalaemia, hyponatraemia, SGLT2 inhibitors

## Abstract

A 36-year-old unmarried male chef was incidentally diagnosed with hypokalemia during an evaluation for an acute perianal abscess. Despite potassium supplementation, he developed progressive weakness in his lower limbs, culminating in an inability to stand. Investigations confirmed severe hypokalemia, metabolic alkalosis, hypomagnesemia, secondary hyperaldosteronism, and low urinary calcium excretion, with normotension. The patient’s long-standing stunted growth and lean physique since childhood were noted. Biochemical assays further identified type 2 diabetes mellitus and metabolic syndrome. Genetic analysis revealed three heterozygous *SLC12A3* mutations (M1: c.421G>A: *p*.G141R, M2: c.509T>A:*p*.L170Q, and M3: c.704C>A: *p*.T235K), compound heterozygo us and derived from both parents, with M1 and M3 reported here for the first time. Treatment with spironolactone and oral potassium chloride stabilized his potassium levels. Following the administration of SGLT2 inhibitors in patients receiving hypoglycemic therapy, we observed a mild decrease in serum sodium levels. This case highlights the criticality of vigilant metabolic surveillance in Gitelman syndrome and advises prudence with SGLT2 inhibitors in those with concurrent type 2 diabetes, given the risk of potentially aggravate sodium loss.

## Introduction

Gitelman syndrome (GS) is an infrequently encountered autosomal recessive condition, originating from mutations in the *SLC12A3* gene encoding the thiazide-sensitive Na-Cl cotransporter. To date, more than 350 mutations distributed across the *SLC12A3* gene have been identified in individuals with Gitelman syndrome (GS). While the majority of GS patients possess compound heterozygous mutations in *SLC12A3* (Blanchard et al., 2017). First described in 1966, GS is characterized by a constellation of symptoms: hypokalemic metabolic alkalosis, hypomagnesemia, hypocalciuria, secondary aldosteronism, with typically normal or low blood pressure. Notably, GS patients are predisposed to significant metabolic challenges.

In our case study, we document a family affected by GS concomitant with metabolic disturbances. Genetic analyses unearthed three pathogenic variants inherited from the patient’s parents, two of which are novel discoveries.

### Patient information

A 36-year-old unmarried male, Han Chinese chef presented to the hospital with a history of abnormal sensations in his limbs persisting for 5 years, limb weakness escalating over 4 months, and a significant deterioration of symptoms over the past day. He described initial symptoms akin to ants crawling and itching on the outer aspect of his left leg, starting 5 years prior without any formal diagnosis or intervention. In the pre-operative evaluation for a perianal abscess 9 days earlier, his blood potassium was critically low at 1.64 mmol/L, with a 24-h urinary potassium output of 27.31 mmol/L. Despite potassium supplementation, his weakness progressed, notably affecting the left lower extremity and impairing his ability to stand. An urgent assessment on 10 March 2022, revealed potassium levels ranging from 1.64 to 2.32 mmol/L, sodium levels from 134.8 to 138.69 mmol/L, chloride levels from 94.91 to 98.86 mmol/L, calcium levels from 1.89 to 2.37 mmol/L, and phosphate levels from 0.62 to 0.79 mmol/L. Furthermore, the patient recounted a history of stunted growth and development from childhood, resulting in a slender and diminutive physique. The patient’s did not know his birth weight; he was delivered at full term via vaginal delivery. His height was 157 cm and weighs 57.5 kg, with a normal BMI 23.3 kg/m^2^, and no reported issues with hearing or vision. His father, with a height of 172 cm and a weight of 83.5 kg, denied a history of diabetes mellitus and hypertension but had a diagnosis of coronary artery disease. His fasting blood glucose level was 6.7 mmol/L, and his potassium level was 3.2 mmol/L (See Table 2). The patient’s mother, 160 cm in height and weighing 65 kg, had hypertension but no history of diabetes mellitus, with normal potassium levels.

Laboratory examinations uncovered severe hypokalemia, elevated urinary potassium (hyperkalemuria), metabolic alkalosis, hypomagnesemia, secondary hyperaldosteronism, and diminished urinary calcium (hypocalciuria) (Table 1). Creatine kinase was found to be elevated at 351.58 U/L, surpassing the normal range of 120–250. Despite these findings, the patient maintained normal blood pressure and had no prior diagnosis of diabetes. Metabolic disturbances were evident, underscored by a glucose tolerance test that revealed modestly elevated postprandial blood glucose levels, a glycated hemoglobin level of 6.8%, and insulin function changes indicative of type 2 diabetes. This was coupled with hyperlipidemia, hyperuricemia, and evidence of mild liver damage (ALT 107.25 U/L, AST 42.74 U/L, AST/ALT ratio 0.58, albumin 42 g/L), as well as fatty liver. Biochemical tests on admission were as Table 1. Kidney ultrasonography showed mild diffuse damage, numerous cysts, and stones, pointing to potential vacuolar degeneration in renal epithelial cells due to prolonged hypokalemia.

**TABLE 1:** Clinical biochemical and metabolic indices of the patient.

Variable	Test	References
	Value	Range
Blood tests		
Potassium (mmol/L)	2.53*	3.5–5.5
Sodium (mmol/L)	138.74	137–147
Chloride (mmol/L)	96.58*	99–110
Calcium (mmol/L)	2.33	2.15–2.55
Magnesium (mmol/L)	0.81	0.66–0.99
Phosphate (mmol/L)	0.62*	0.85–1.51
Arterial blood gas analysis		
PH	7.47*	7.35–7.45
PO2 (mmHg)	96.8	83–108
PCO2 (mmHg)	33.4*	35–45
Bicarbonate (mmol/L)	27.4*	18–23
Renin–angiotensin–aldosterone system		
Angiotensin II (pg/mL) (supine)	271.8*	10–160
Plasma renin activity (ng/mL/h) (supine)	780.01*	4–24
Plasma aldosterone (ng/mL) (supine)	162.34*	25–129
Angiotensin II (pg/mL) (upright)	371.08*	40–310
Plasma renin activity (ng/mL/h) (upright)	1388.71*	4–38
Plasma aldosterone (ng/mL) (upright)	165.75	49–252
24-h urine tests		
Potassium (mmol/24 h)	144.25*	25–125
Calcium (mmol/24 h)	1.36*	2.5–7.5
Sodium (mmol/24 h)	213.59	130–260
Phosphate (mmol/24 h)	27.81	22–48
Magnesium (mmol/24 h)	2.49*	3.0–4.5
Metabolic factors		
ALT (IU/L)	107.25*	9–50
AST (U/L)	42.74*	15–40
r-GT (U/L)	221.87*	10–60
Glycosylated hemoglobin, %	6.8*	3.9–6.0
CK(U/L)	351.58*	50–310
Total cholesterol (mmol/L)	6.37*	2.9–5.17
Triglyceride (mmol/L)	3.7*	0.22–1.7
LDL-c (mmol/L)	4.14*	0–3.36
HDL-c (mmol/L)	1.13	0.9–2.19
blood uric acid (*μ* mol/L)	515*	208–428

**FIGURE 1:**
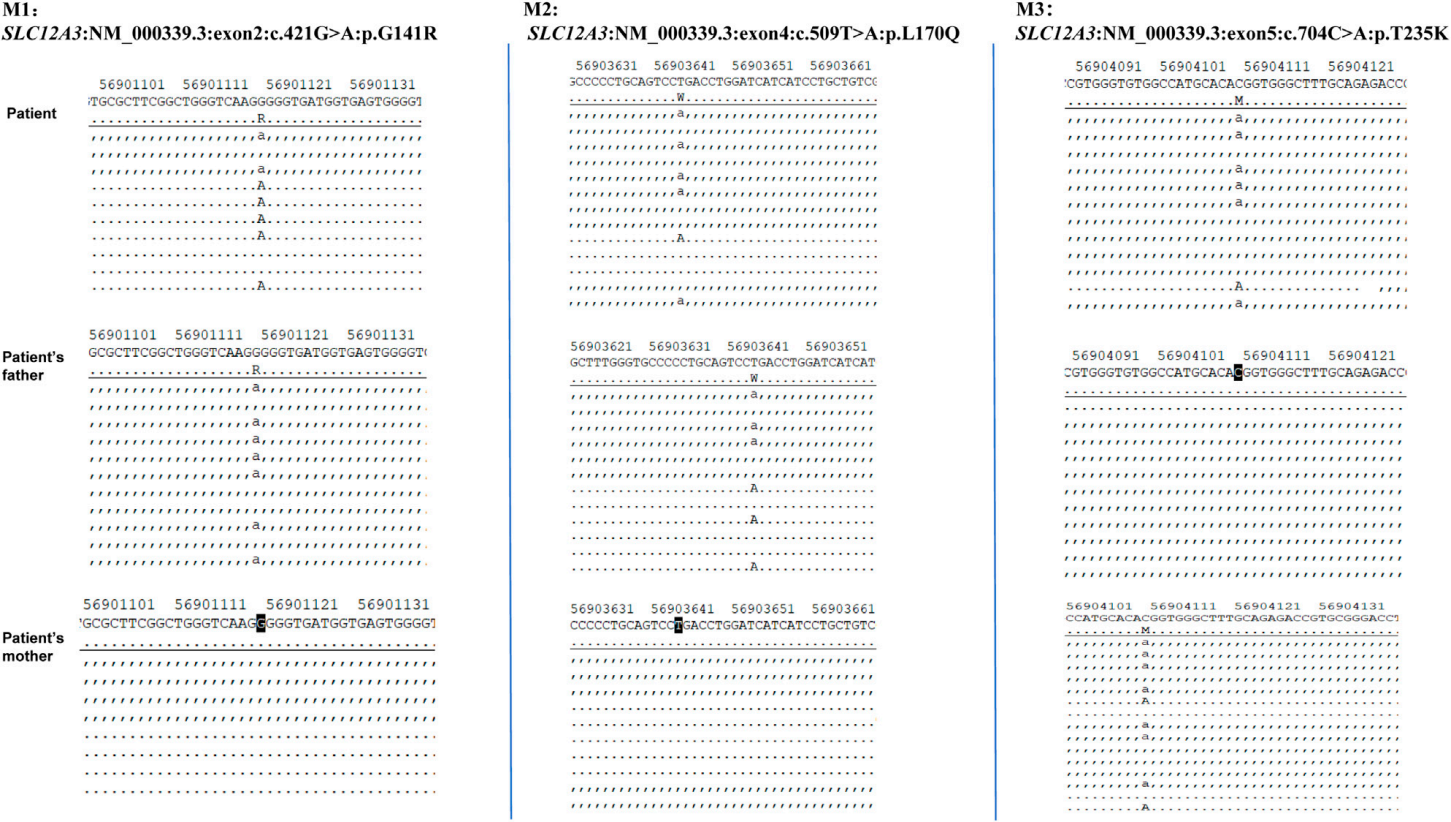
Genetic analysis of SLC12A3 mutations in a Gitelman syndrome family trio.

**FIGURE 2:**
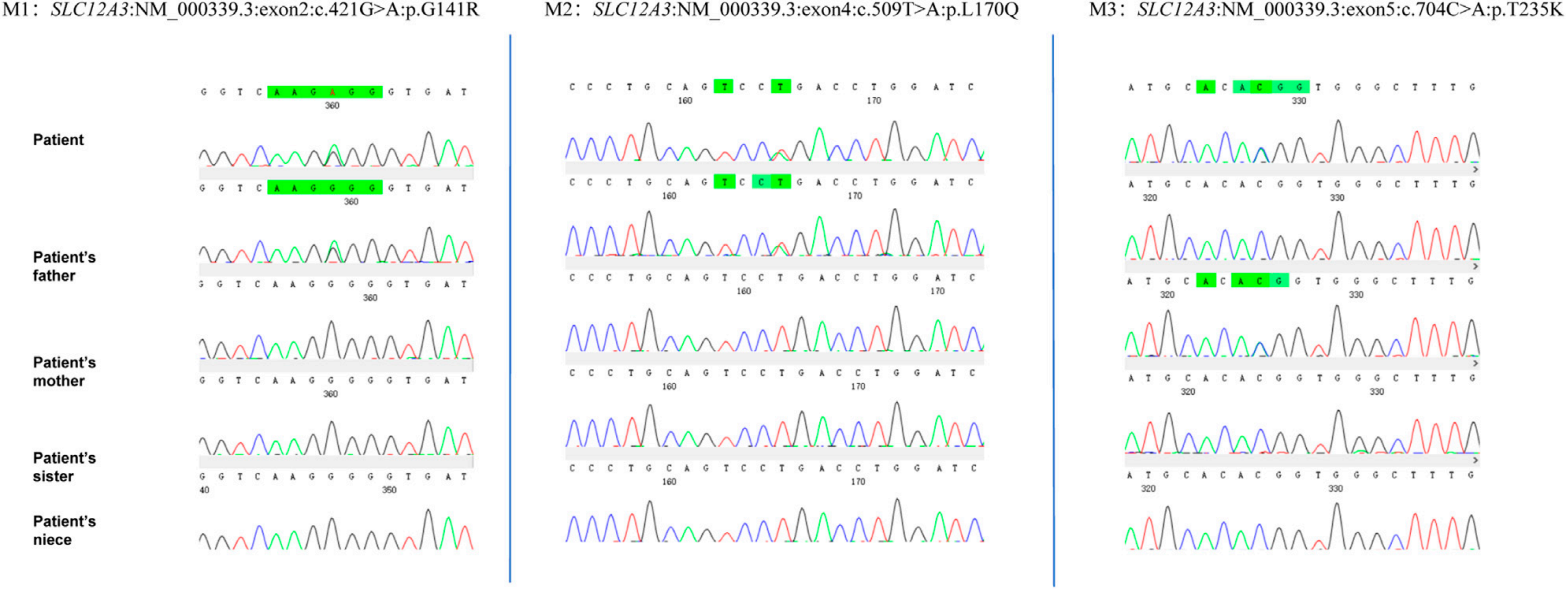
The proband’s next-generation sequencing (NGS) analysis identified three heterozygous variants in the SLC12A3 gene: a missense variant (c.421G>A) resulting in the amino acid substitution p.G141R, and a second variant (c.509T>A and c.704C>A) leading to the amino acid changes p.L170Q and p.T235K, respectively. These genetic findings were confirmed by Sanger sequencing, as depicted in the electropherograms.

**FIGURE 3:**
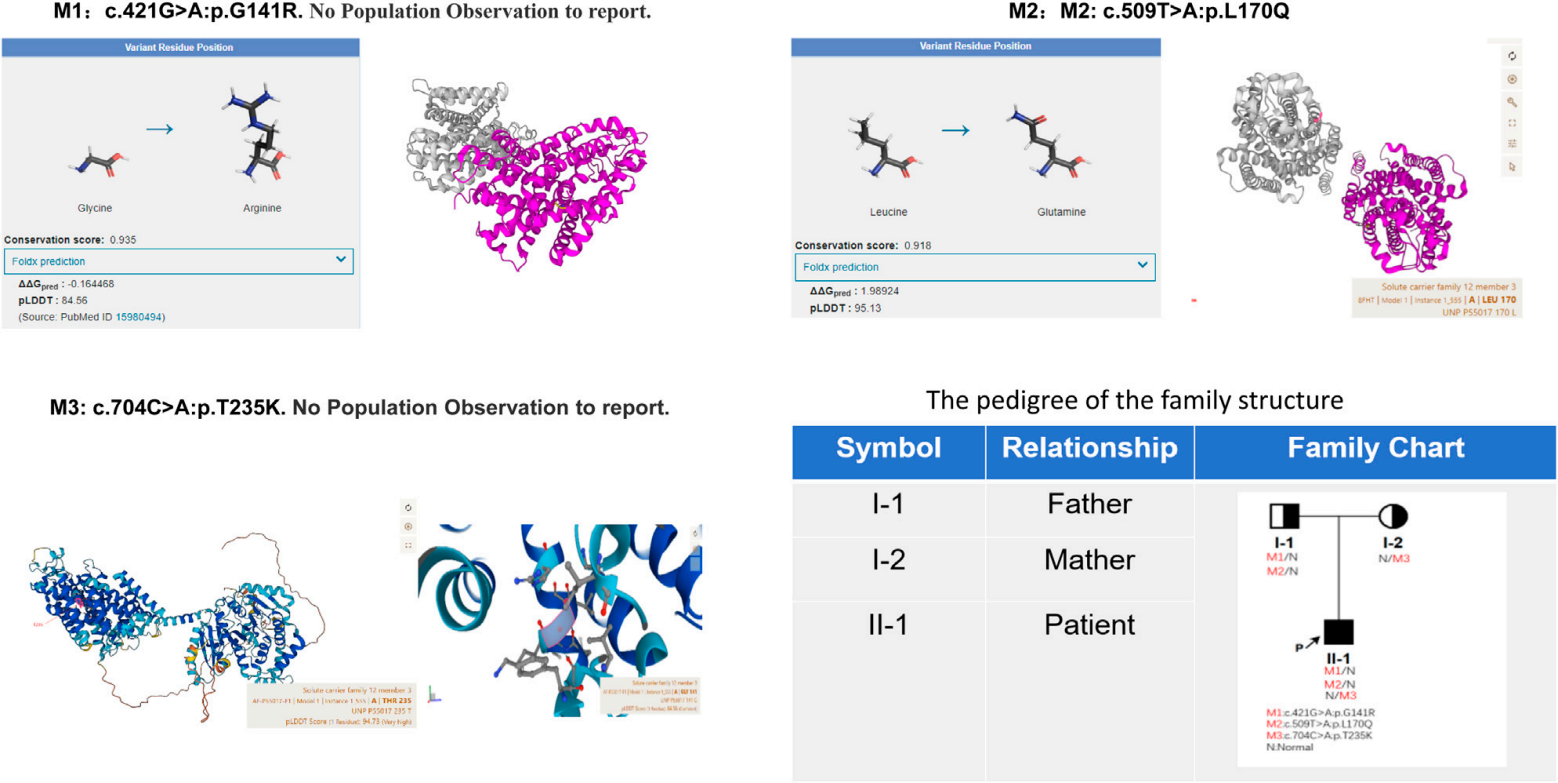
The protein encoded by the M2 mutation site is highly conserved (Conservation score: 0.918) and plays an important role in protein function; and the mutation reduces protein stability (Foldx prediction 1.98924). AlphaFold AI system helps us to predict the mutation protein’s 3D structure (https://alphafold.ebi.ac.uk/). The family pedigree provided illustrates the distribution of these *SLC12A3* variants among the members. Individuals carrying the compound heterozygous variants are indicated with marked symbols. Circles represent female family members, and squares represent male family members, with the arrow pointing to the proband. The patient’s father carries M1 and M2 mutation alleles, presenting with mild hypokalemia but without clinical symptoms, and has no diabetes. The mother carries the M3 mutation yet exhibits no clinical symptoms.

Given the patient’s symptoms and lab results, Gitelman syndrome (GS) was suspected, leading to genetic analysis of both the patient and his relatives. Next-generation sequencing (NGS) uncovered three heterozygous missense mutations in the *SLC12A3* gene: M1: c.421G>A: *p*.G141R, M2: c.509T>A: *p*.L170Q, and M3: c.704C>A: *p*.T235K (Figure 1). Specifically, M1 causes a glycine-to-arginine swap at amino acid position 141, M2 changes leucine to glutamine at position 170, and M3 alters threonine to lysine at position 235. These mutations were subsequently confirmed by Sanger sequencing, attesting to their authenticity (Figure 2). Further familial investigation indicated the M1 and M2 mutations were derived from the father, who exhibited mild hypokalemia without diabetes, symptoms, or hypertension (Table 2). The M3 mutation traced back to the mother, with all her relevant health markers within normal range. These mutations constitute rare compound heterozygous variations (Figure 3). Importantly, while mutation M2 is recorded in the Human Gene Mutation Database (HGMD) and ClinVar, mutations M1 and M3 are newly identified in this context.

**TABLE 2:** Father’s electrolytes results.

Arterial blood gas analysis		
PH	7.38	7.35–7.45
cHCO3-(mmol/L)	28.2	22.5–26.9
SBE,c (mmol/L)	7.0*	−3.0–2.0
Bicarbonate (mmol/L)	29	20–31
Blood tests		
Potassium (mmol/L)	3.32*	3.5–5.5
Sodium (mmol/L)	140.64	137–147
Chloride (mmol/L)	101.78	99–110
Calcium (mmol/L)	2.28	2.15–2.55
Magnesium (mmol/L)	0.8	0.66–0.99
Phosphate (mmol/L)	1.14	0.85–1.51
Subsequent urine		
Potassium (mmol/L)	57.76*	10.25–52.76
Sodium (mmol/L)	49.94	40–222
Chloride (mmol/L)	61.4	25–221
Calcium (mmol/L)	1.82	0.48–5.87
Phosphate (mmol/L)	19.41	3.59–26.36
Magnesium (mmol/L)	2.94	0.5–4.01
Urine Creatinine (μmol/L)	17,388.24	

**FIGURE 4:**
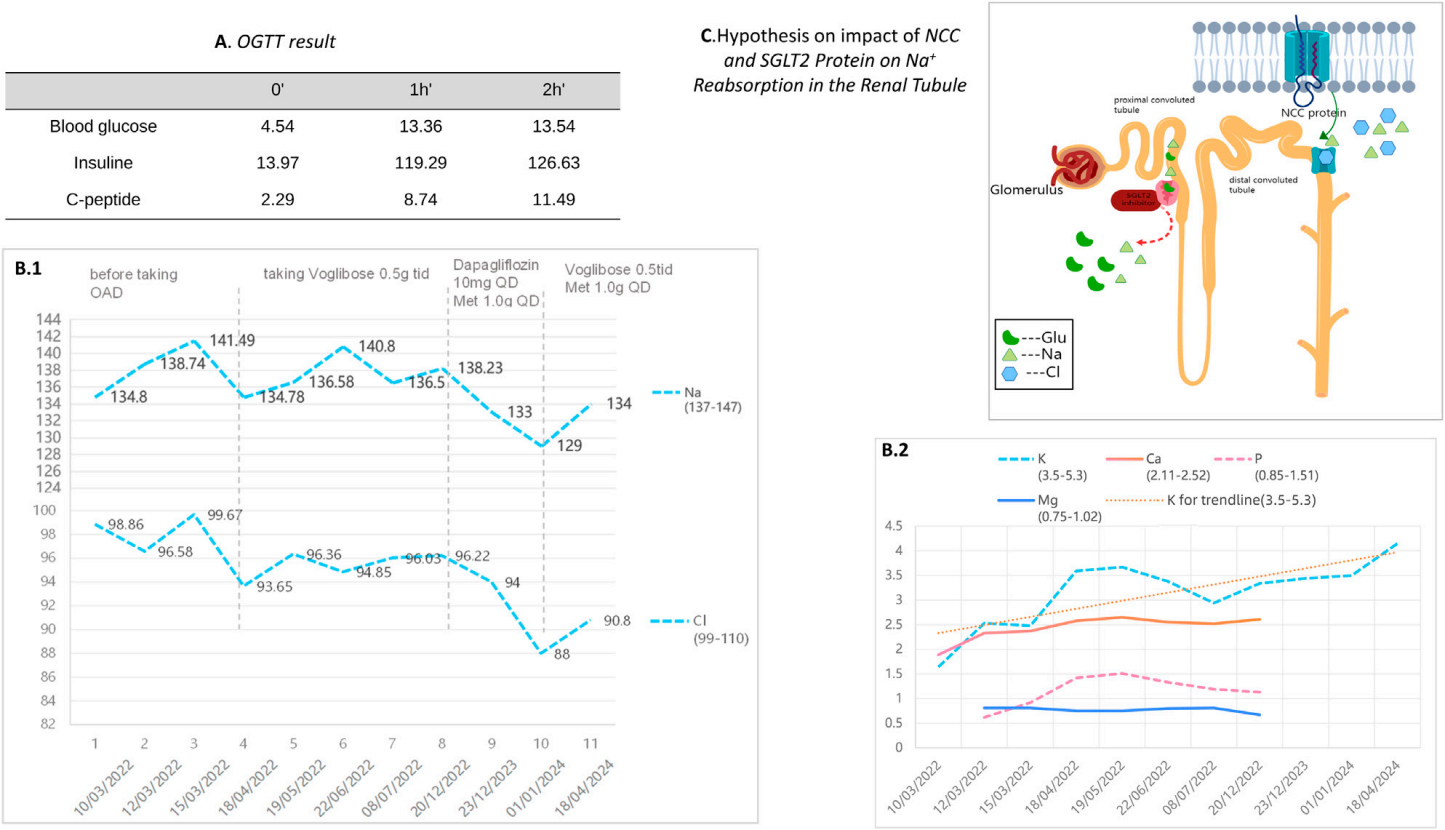
**(A)** presents the results of the patient’s Oral Glucose Tolerance Test (OGTT). **(B.1)** and **(B.2)** depict the changes in electrolyte levels before and after the adjustment of hypoglycemic medication during a two-year follow-up period for our patient. **(C)** illustrates a schematic of the ion regulation by the NCC and SGLT2 proteins in the renal tubules. The NCC protein reabsorbs sodium and chloride in renal tubules. When NCC function is impaired, compensatory mechanisms in other renal segments enhance sodium reabsorption to maintain blood volume and pressure. The effect of SGLT2 inhibitors on NCC’s ion regulation during glucose lowering remains unclear.

Ultimately, the integration of clinical observations and genetic findings led to the diagnosis of Gitelman syndrome (GS) alongside type 2 diabetes mellitus (T2DM) in the patient (Figure 4A). The simultaneous identification of diabetes and the inheritance of two mutations from the father highlights the significance of familial medical history in understanding GS, as well as illuminating the genetic intricacies inherent to this disorder.

### Treatment

The patient underwent a holistic treatment regimen encompassing antidiabetic, lipid-lowering, and uric acid-reducing therapies, alongside supplementation with potassium and magnesium, and the administration of aldosterone antagonists. This multifaceted approach effectively normalized the patient’s blood potassium and glucose concentrations. ARBs were once tried on the patient as one of the options for combination medication but were discontinued because of hypotension. Afterwards, oral spironolactone (thrice daily, three tablets per dose) and chloride (KCl) (thrice daily, three tablets per dose) have successfully maintained potassium levels within a healthy range. Subsequently, potassium control was achieved with spironolactone and potassium chloride tablets alone, hence NSAIDs (COX) medications had not been tried further.

To manage type 2 diabetes, an alpha-glucosidase inhibitor (AGI) was first prescribed, leading to a glycated hemoglobin level of 6.9% at the 3-month mark. Treatment was then modified to include 10 mg of dapagliflozin and 1.0g of metformin daily, as administered by a local hospital. Ongoing monitoring over the past year revealed potassium levels consistently between 3.75 and 4.06 mmol/L, fasting blood glucose at 6.35 mmol/L, and glycated hemoglobin at 6.2%. It is noteworthy that the combined use of an SGLT-2 inhibitor and spironolactone did not impact the patient’s potassium levels adversely; however, we observed a trend of mild hyponatremia, with sodium levels ranging from 129–136 mmol/L, while urinary sodium remained normal. After the patient stopped using SGLT2i and switched back to metformin in combination with acarbose, the patient’s blood sodium levels rebounded (Table 3).

**TABLE 3:** Patient’s electrolyte follow-up results and medication details.

Medication details	Date	Serum (mmol/L)						Date	Random urine						
		K (3.5–5.3)	Na (137–147)	Cl (99–110)	Ca (2.11–2.52)	*p* (0.85–1.51)	Mg (0.75–1.02)		K (mmol/L) (10.25–52.76)	Na (mmol/L) (40–222)	Cl (mmol/L) (25–221)	Ca (mmol/L) (0.48–5.87)	*p* (mmol/L) (3.59–26.36)	Mg (mmol/L) (0.5–4.01)	Urine creatine (*μ* rine)
No taking medication	20220310	1.64	134.8	98.86	1.89										
No taking medication	20220312	2.53	138.74	96.58	2.33	0.62	0.81								
only high-salt diet and potassium supplementation	20220315	2.48	141.49	99.67	2.37	0.92	0.81	20220315	56.95	52.88	84.23	0.36	15.09	1.03	3234.96
KCl 3 tablets tid, spironolactone 3 tablets tid.Voglibose 1 tablets tid	20220418	3.59	134.78	93.65	2.58	1.42	0.75	20220418	48.79	89.13	109.27	0.66	7.98	1.56	5191.28
Voglibose、spironolactone and KCL same dose	20220519	3.67	136.58	96.36	2.65	1.51	0.75								
Same	20220622	3.38	140.8	94.85	2.55	1.33	0.8								
The patient self-reduced the dose of spironolactone from 3 tablets tid to 2 tablets tid, then returned to the original dosage	20220708	2.94	136.5	96.03	2.52	1.19	0.81								
Switched from AGI to SGLT2	20221220	3.34	138.23	96.22	2.61	1.13	0.67								
SGLT2	20231126	3.44	133	94											
SGLT2	20240101	3.5	129	88											
switched to 2 tablets of metformin once daily and 1 tablet of acarbose three times a day	20240418	4.15	134	90.8	2.62	1.21	0.69								

## Discussion

Gitelman syndrome (GS) is a rare hereditary disorder, affecting roughly one in 40,000 people globally. Nonetheless, research suggests a heightened prevalence in East Asian populations (Tago et al., 2004; Hsu et al., 2009), with estimates of 2.3 and 1.9 per 1,000 individuals according to the HGVD and jMorp databases, respectively. This discrepancy underscores the potential ethnic variability in GS incidence. Typically manifesting in adolescence or early adulthood, GS is noted for its clinical and genetic diversity. Clinically, it presents with a spectrum of hypokalemia severity, whereas genetically, it exhibits variability that may account for the observed ethnic differences in incidence rates.

Gitelman syndrome (GS) is primarily caused by mutations in the *SLC12A3* gene, leading to impaired function of the sodium-chloride cotransporter (NCC) (Viering et al., 2022). This transporter is pivotal for the reabsorption of sodium and chloride ions within the distal convoluted tubule of the kidney, thereby playing an essential role in managing renal electrolyte balance and regulating blood pressure (Richardson et al., 2008; Cruz-Rangel et al., 2011; Glaudemans et al., 2012; Nan et al., 2022; Fan et al., 2023). Furthermore, the NCC protein participates in the signaling pathways of various pro-inflammatory cytokines, including IL-18 and IL-6 (Wang et al., 2015; Wynne et al., 2022). Such involvement underscores the significance of the NCC protein not only in preserving electrolyte homeostasis and blood pressure but also in mediating the body’s inflammatory responses. This suggests that the repercussions of NCC dysfunction extend beyond mere electrolyte imbalances, implicating broader physiological and possibly pathological processes.

The management of Gitelman Syndrome (GS) is predominantly aimed at alleviating symptoms and rectifying electrolyte imbalances through the supplementation of potassium and magnesium, alongside the employment of ACE inhibitors (ACEIs)/angiotensin receptor blockers (ARBs) and aldosterone antagonists. Notably, GS patients exhibit a higher incidence of diabetes compared to the general population, with glucose metabolism disturbances mainly manifesting as insulin resistance and impaired fasting glucose (Yuan et al., 2017; Yin et al., 2023). These issues are thought to arise from hypokalemia’s detrimental impact on pancreatic β-cell responsiveness to hyperglycemia and its interference with insulin secretion (Sun et al., 2014; Zhang et al., 2018; Hall et al., 2020). Additionally, hypomagnesemia may impair the tyrosine kinase activity within insulin receptors, further complicating glucose management (Liu et al., 2021).

In this case report, the proband was only 36 years old but presented with concurrent diabetes and metabolic dysregulation, signifying an early onset. We posit the following considerations: on one hand, approximately 11% of Chinese have diabetes based on the data from the latest epidemiological study in China, and young-onset diabetes is becoming increasingly common (Xu et al., 2013; Ma, 2018). On the other hand, considering that chronic hypokalemia can affect insulin secretion and exacerbate glucose abnormalities, the patient’s long-standing low potassium levels, which remained uncorrected, could have compounded the condition (Komada et al., 2020).

At present, there’s a lack of consensus regarding the optimal diabetic medication for individuals with GS, with insufficient data on the potential adverse effects of various hypoglycemic agents on this condition. Research indicates that SGLT2 inhibitors could potentially reduce hyperkalemia incidence without exacerbating hypokalemia (Ferreira et al., 2022; Edwards et al., 2023). SGLT2 inhibitors are recognized for their cardioprotective and nephroprotective properties, thus making them particularly advantageous for patients at high risk of cardiovascular diseases and those suffering from chronic kidney disease (Zelniker et al., 2019; Rahman et al., 2023). Recent studies highlight that the administration of SGLT2 inhibitors can enhance magnesium balance in diabetic patients (Ray, 2020). However, in this particular case, no restoration of serum magnesium levels was observed after the administration of SGLT2-i in GS patient. Reports on the choice of hypoglycemic drug in GS patients are scarce, and some case studies suggest that SGLT2 inhibitors might lead to exacerbated hypotension and heightened diabetic ketoacidosis risk in their case (Ahmed et al., 2021; Yang et al., 2023). The specific influence of SGLT2 inhibitors on GS-related electrolyte fluctuations remains unreported, indicating a gap in current medical knowledge and underscoring the need for further investigation.

The Alpha-glucosidase inhibitor (AGI) was initially chosen for its mild antihyperglycemic properties. However, after identifying inadequate glycemic control during a follow-up at a local hospital, the patient was switched to dapagliflozin. Continuous monitoring thereafter indicated the onset of hyponatremia, though blood pressure remained within normal limits, subsequent to the SGLT2 inhibitor’s administration. The patient experienced a mild decrease in blood sodium levels after switching from AGI to SGLT2 inhibitors on a regimen of chronic potassium chloride supplementation and spironolactone use. The hyponatremia symptoms improved after discontinuing the medication (Table 3). Additionally, the patient developed mild gynecomastia after long-term oral administration of spironolactone. The patient attempted to reduce the spironolactone dose to 2 tablets taken three times daily, which led to a temporary drop in serum potassium to 2.94 mmol/L. After increasing the spironolactone back to 3 tablets three times daily, the serum potassium levels returned to normal. The patient currently finds this tolerable. We analyze how SGLT2 inhibitors might contribute to hyponatremia, supported by an attached schematic (Figures 4B, 4C). This development could be linked to GS patients’ characteristic NCC protein dysfunction, which demands precise blood sodium level regulation. Incorporating an SGLT2 inhibitor into the therapeutic approach might exacerbate pre-existing electrolyte imbalances, particularly increasing the risk of both hyponatremia and hypokalemia.

Patients with Gitelman Syndrome (GS) should be mindful of their salt intake—high salt diet, to ensure their serum sodium levels remain within a normal range (Blanchard et al., 2017). Typically, GS patients have normal sodium levels due to compensatory mechanisms such as the activation of the Renin-Angiotensin System (RAS) and a dilution effect, which help regulate and maintain sodium balance. This ability to maintain normal blood pressure and sodium levels positions GS as a fascinating natural counterpart for studying hypertension (Fan et al., 2023).

In this particular case, the patient was treated with an ARB medication, losartan, combined with spironolactone. Although this regimen successfully maintained potassium levels, it led to notable hypotension, indicating that ARB medications might not be appropriate for all individuals with Gitelman Syndrome (GS). The observed hypotension may result from excessive suppression of the Renin-Angiotensin System (RAS) compensatory pathways, thereby destabilizing the body’s electrolyte equilibrium. This theory warrants additional investigation through both experimental studies and clinical observations to be substantiated.

Moreover, research suggests that the electrolyte changes observed in GS, coupled with pronounced RAS activation without accompanying high blood pressure or cardiac remodeling, offer a unique human model for exploring cardiovascular and renal remodeling as well as the RAS pathway and oxidative stress. The electrolyte shifts in GS also hint at the kidneys’ potential to employ additional regulatory mechanisms under various pathological states to maintain electrolyte balance. Specifically, when transporter protein function is compromised, other renal segments, such as the proximal convoluted tubules and collecting ducts, may compensate by adjusting sodium and water reabsorption to preserve overall balance.

## Conclusion

This study highlights the identification of two novel mutations within the *SLC12A3* gene [c.421G>A: *p*.G141R and c.704C>A: *p*.T235K], contributing to the broader understanding of Gitelman Syndrome’s genetic diversity. In managing diabetes among GS patients, meticulous monitoring of electrolyte concentrations, particularly sodium and potassium levels, is imperative when employing SGLT2 inhibitors. Furthermore, individuals with GS must exercise caution when utilizing ACE inhibitors or ARBs to circumvent potential issues such as hypotension and disturbances in electrolyte homeostasis.

## Data Availability

The datasets presented in this study can be found in online repositories. This data can be found here in ClinVar using accession number SCV005061484.
